# Supplementary material for: Weakened APC/C activity at mitotic exit drives cancer vulnerability to KIF18A inhibition
Source: EMBO J. 2024 Jan 26;43(5):2. doi: 10.1038/s44318-024-00031-6 (PMC10907621; doi:10.1038/s44318-024-00031-6)
Supplement: Supplementary file 1 — Appendix [file 44318_2024_31_MOESM1_ESM.pdf]

Appendix Figures

Figure Name	Page
Appendix Figure S1: Validation of OVCAR-3 and HCC1806 CRISPR Cas9 screen hits	2
Appendix Figure S2: Extended SAC KIF18Ai rescue data	3
Appendix Figure S3: Extended data for photoactivatable GFP-α-Tubulin cell lines	4
Appendix Figure S4: Extended data for WGD cell lines	5
Appendix Figure S5: Validation of OVCAR-8 CRISPR Cas9 screen hits	6
Appendix Figure S6: Validation of APC/C activated cell lines	7

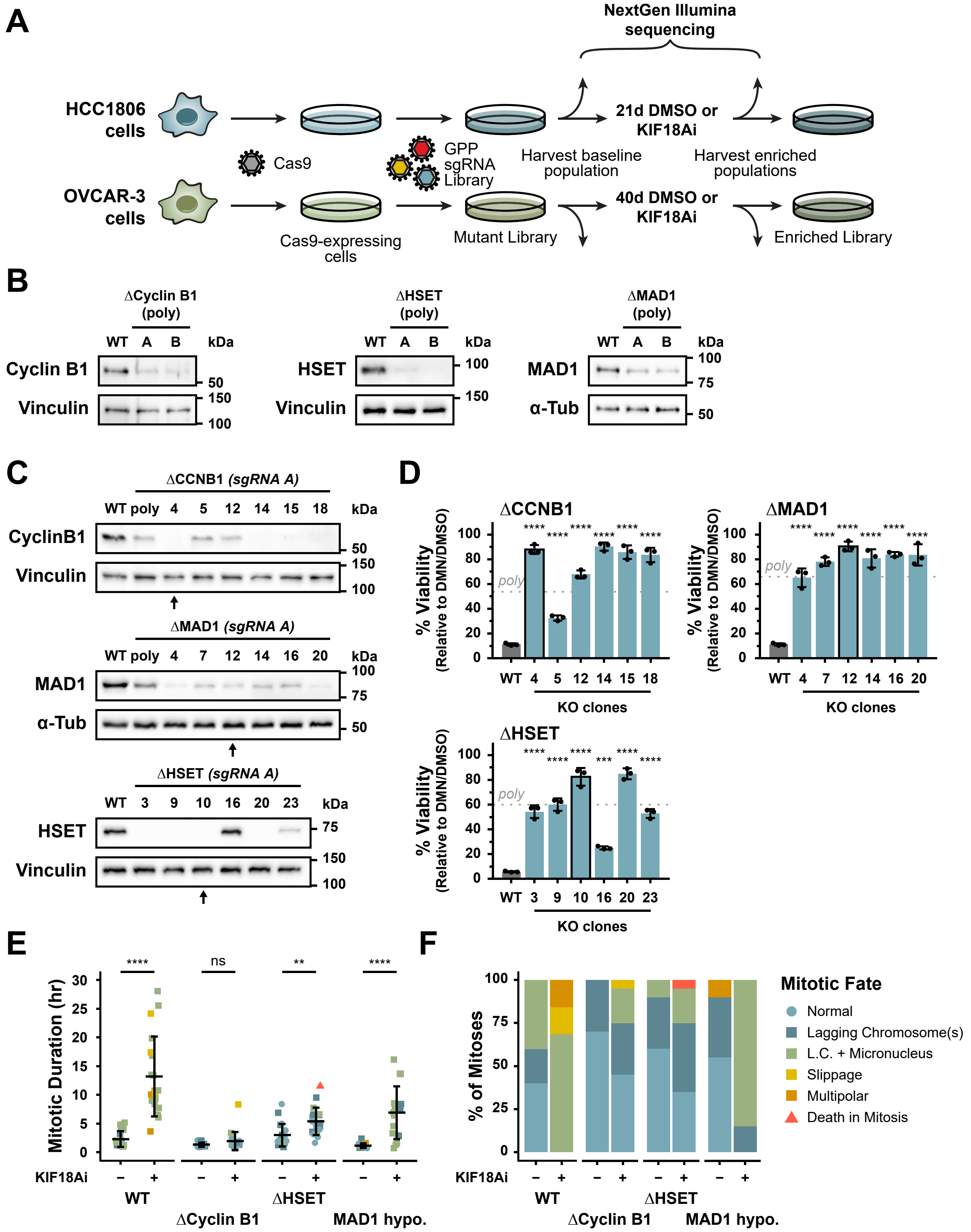

Appendix Figure S1: Validation of OVCAR-3 and HCC1806 CRISPR Cas9 screen hits

(A) Schematic of whole-genome CRISPR-Cas9 knockout screen protocol. **(B)** Western blot validation of polyclonal HCC1806 sgRNA-targeted cell lines for Cyclin B1, HSET, and MAD1. **(C)** Western blot validation of clonal HCC1806 sgRNA-targeted cell lines for Cyclin B1, HSET, and MAD1. Arrow denotes the clone used in further experiments. **(D)** 5-day MTT endpoint viability assay of HCC1806 clonal CRISPR-Cas9 KO cell lines in KIF18Ai. Data are represented as mean ± SD. N = 3 technical replicates. Boxed bar denotes the clone used in further experiments. Statistical significance was determined using a one-way-ANOVA with post-hoc Dunnett's multiple comparisons test between each set of clonal edited cells and WT. Full statistical results are listed in Dataset EV3. **(E)** Quantification of live-cell widefield timelapse microscopy of H2B/α-Tubulin fluorescently tagged edited cell lines colored by mitotic outcome. Error bars represent mean ± SD. Statistical significance was determined using an unpaired two-tailed Student's t-test. Sample size and full statistical results are listed in Dataset EV3. **(F)** Proportion of mitotic fates across the panel of H2B/α-Tubulin fluorescently tagged HCC1806 rescue cell lines in response to DMSO or KIF18Ai treatment from live-cell widefield timelapse microscopy.*$P < 0.05$, **$P < 0.01$, ***$P < 0.001$ and ****$P < 0.0001$ (D–E).

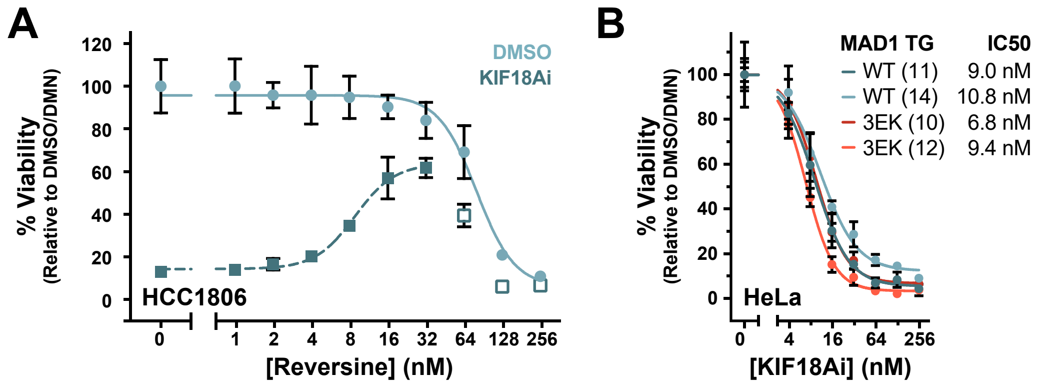

Appendix Figure S2: Extended SAC KIF18Ai rescue data

(A) Titration of MPS1 inhibitor Reversine in a 5-day MTT endpoint viability assay against DMSO or KIF18Ai treated HCC1806 cells. Open squares are omitted from the curve fit. N = 3 technical replicates from a single experiment. Data are represented as mean ± SD. **(B)** Titration of KIF18Ai in 5-day MTT endpoint viability assay for HeLa MAD1 knockout FRT TetON VSV-MAD1 and Cyclin B1-binding deficient HeLa MAD1 knockout FRT TetON VSV-MAD1(E52K, E53K, E56K) cell lines. N = 3 technical replicates from single experiment. Data are represented as mean ± SD.

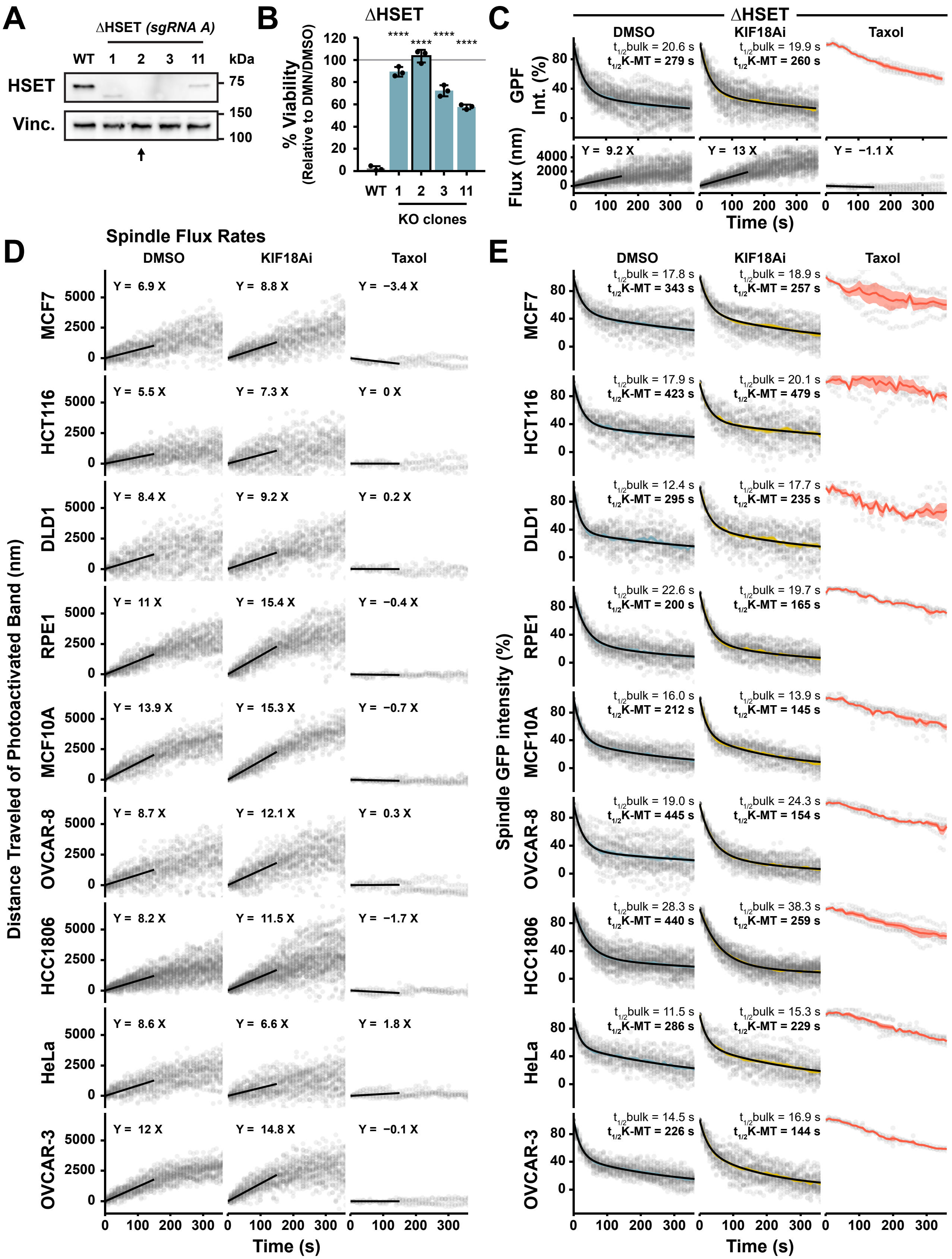

Appendix Figure S3: Extended data for photoactivatable GFP-α-Tubulin cell lines

(A) Western blot validation of clonal HCC1806 H2B-iRFP PA-GFP-α-Tubulin HSET knockout cell lines. Arrow denotes preferred clone. **(B)** 5-day MTT endpoint viability assay of clonal HCC1806 H2B-iRFP PA-GFP-α-Tubulin HSET knockout cell lines in KIF18Ai. Data are represented as mean ± SD. N = 3 technical replicates from single experiment. Boxed bar denotes preferred clone. Statistical significance was determined using a one-way-ANOVA with post-hoc Dunnett's multiple comparisons test between each set of clonal edited cells and WT. Full statistical results are listed in Dataset EV3. *$P < 0.05$, **$P < 0.01$, ***$P < 0.001$ and ****$P < 0.0001$. **(C)** Top: Two-phase exponential decay fit of photoactivated spindle from HCC1806 PA-GFP-α-Tubulin ΔHSET cells. The shaded colored region represents mean ± SEM. Gray dots represent individual measurements. Bottom: Spindle flux rate of photoactivated band from movies. Linear fit is calculated from data between 0-150 s. N > 50 cells/condition. **(D)** Spindle flux rate of photoactivated band from live-cell confocal timelapse movies of cell line panel as in C. N ≥ 30 cells/condition. **(E)** Two-phase exponential decay fit of integrated intensity measurements of the mitotic spindle after photoactivation from live-cell confocal timelapse movies of cell line panel as in C. N ≥ 30 cells/condition.

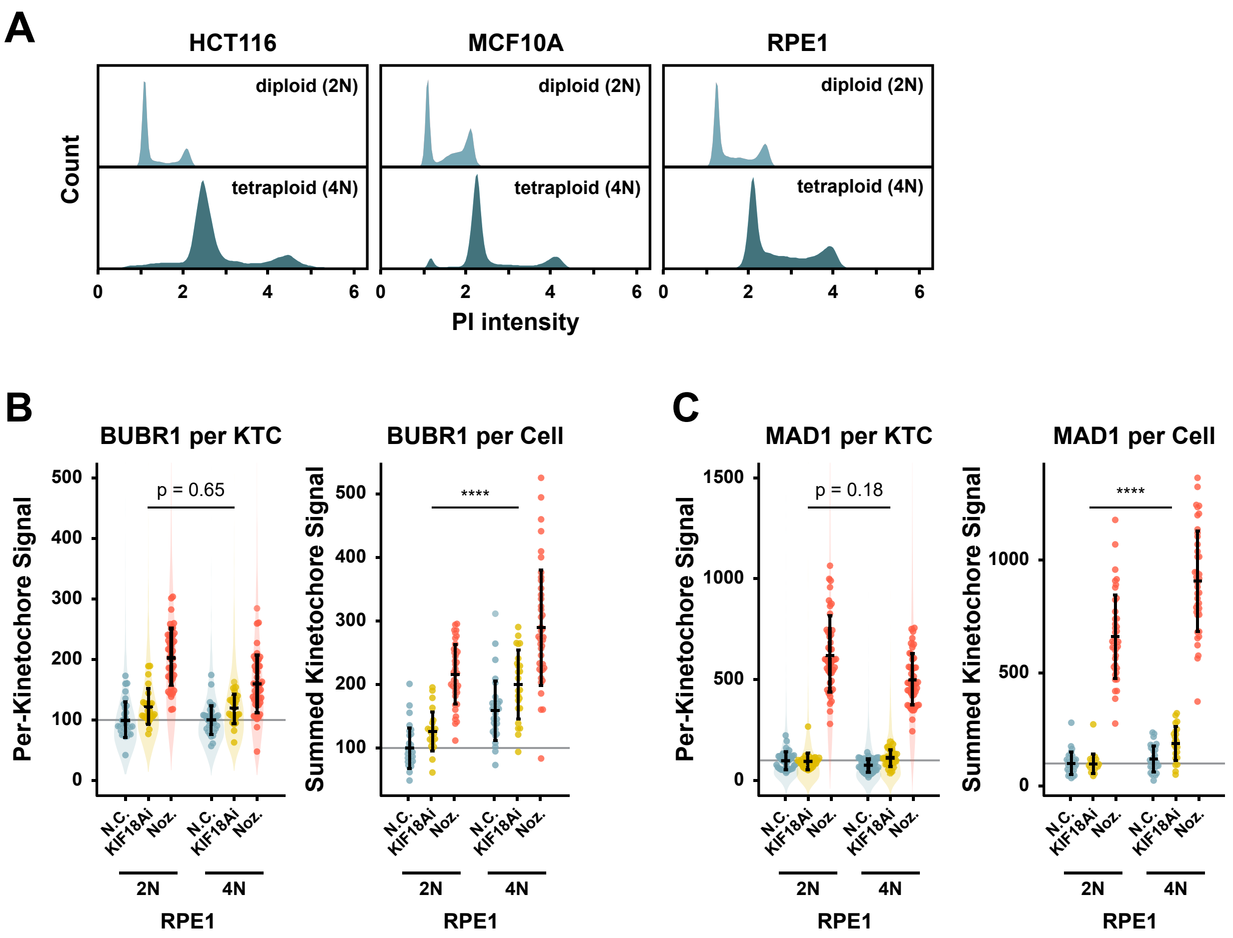

Appendix Figure S4: Extended data for WGD cell lines

(A) Propidium iodide flow cytometry ploidy analysis of diploid and WGD HCT116, MCF10A, and RPE1 cell lines. N ≥ 5000 cells per sample. **(B)** Intensity of BUBR1 at kinetochores in DMSO, KIF18Ai, and Nocodazole treatments between diploid and WGD RPE1 cells from wide-field immunofluorescence images. Left: intensity at individual kinetochores. Right: summed kinetochore intensity per cell. Violin plots summarize all individual kinetochores analyzed, points represent per-cell intensity measurements, and error bars are mean ± SD of per-cell measurements. Statistical significance was determined using an unpaired two-tailed Student's t-test between 2N and 4N KIF18Ai conditions. Sample size and full statistical results are listed in Dataset EV3. **(C)** Intensity of MAD1 at kinetochores in DMSO, KIF18Ai, and Nocodazole treatments between diploid and WGD RPE1 cells from wide-field immunofluorescence images. Left: intensity at individual kinetochores. Right: summed intensity per cell. Violin plots summarize all individual kinetochores analyzed, points represent per-cell intensity measurements, and error bars are mean ± SD of per cell measurements. Statistical significance was determined using an unpaired two-tailed Student's t-test between 2N and 4N KIF18Ai conditions. Sample size and full statistical results are listed in Dataset EV3. *P < 0.05, **P < 0.01, ***P < 0.001 and ****P < 0.0001 (B-C).

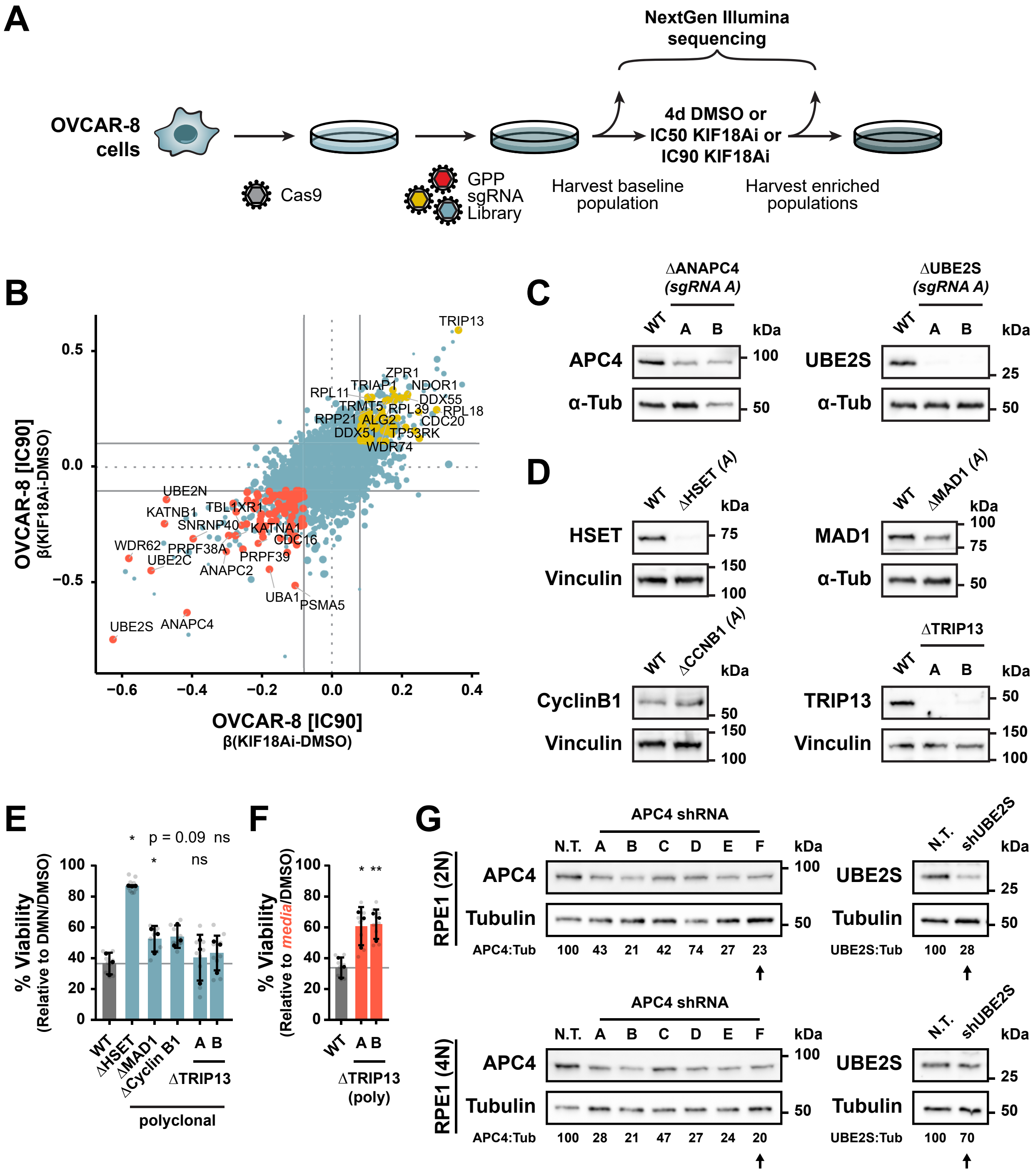

Appendix Figure S5: Validation of OVCAR-8 CRISPR Cas9 screen hits

(A) Schematic of whole-genome CRISPR-Cas9 knockout screen protocol. **(B)** Comparison of OVCAR-8 whole genome CRISPR-Cas9 screens with KIF18Ai at IC50 and IC90 concentrations. Data is annotated with mean ± 1.5 × SD. Genes whose loss is selected against in KIF18Ai with an average FDR < 0.1 are highlighted in red. Genes whose loss grants a growth advantage in KIF18Ai with an average FDR < 0.1 are highlighted in yellow. **(C)** Western blot validation of polyclonal OVCAR-8 sgRNA-targeted cell lines for APC4 and UBE2S. **(D)** Western blot validation of polyclonal OVCAR-8 sgRNA-targeted cell lines for HSET, MAD1, Cyclin B1, and TRIP13. **(E)** 5-day MTT endpoint viability assay of polyclonal OVCAR-8 edited cell lines in KIF18Ai. Data are represented as mean ± SD. N = 3 independent experiments, n = 3 technical replicates per experiment. Statistical significance was determined using a one-way-ANOVA with post-hoc Dunnett's multiple comparisons test between edited lines and WT. Full statistical results are listed in Dataset EV3. **(F)** Media normalized 5-day MTT endpoint viability assay of polyclonal OVCAR-8 TRIP13 knockout cell lines in KIF18Ai. Media normalization was used rather than DMN normalization since TRIP13 knockout generated growth rescue in DMN. Data are represented as mean ± SD. N = 3 independent experiments, n = 3 technical replicates per experiment. Statistical significance was determined using a one-way-ANOVA with post-hoc Dunnett's multiple comparisons test between edited lines and WT. Full statistical results are listed in Dataset EV3. **(G)** Western blot validation of APC4 and UBE2S knockdown in polyclonal RPE1 diploid and tetraploid cell lines. Arrows indicate the cell line used for unique shRNA experiments. *P < 0.05, **P < 0.01, ***P < 0.001 and ****P < 0.0001 (E-F).

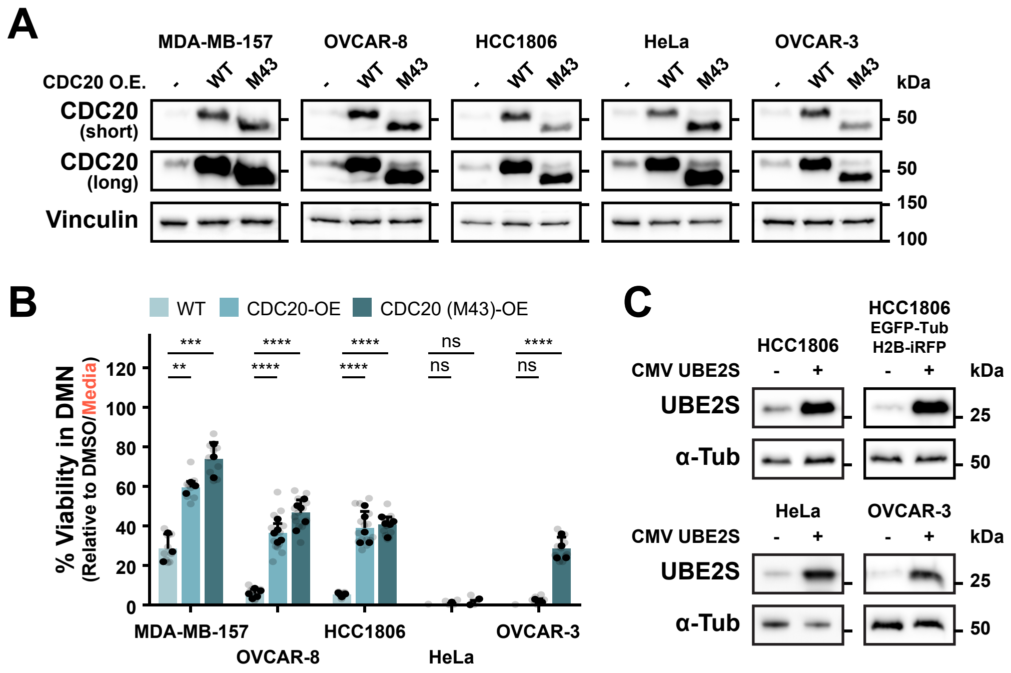

Appendix Figure S6: Validation of APC/C activated cell lines

(A) Western blot validation of polyclonal cell lines overexpressing the WT and M43 translational isoform of CDC20. **(B)** Media normalized 5-day MTT endpoint viability assay of sensitive cell lines overexpressing either WT or M43 CDC20 in DMN. Data are represented as mean ± SD. N ≥ 3 independent experiments per cell line, n = 3 technical replicates per experiment. Statistical significance was determined using a one-way-ANOVA with post-hoc Dunnett's multiple comparisons test between each overexpression pair and WT. Full statistical results are listed in Dataset EV3. *P < 0.05, **P < 0.01, ***P < 0.001 and ****P < 0.0001. **(C)** Western blot validation of polyclonal cell lines overexpressing UBE2S.
