# Supplementary material for: Weakened APC/C activity at mitotic exit drives cancer vulnerability to KIF18A inhibition
Source: EMBO J. 2024 Jan 26;43(5):2. doi: 10.1038/s44318-024-00031-6 (PMC10907621; doi:10.1038/s44318-024-00031-6)
Supplement: Supplementary file 8 — Movie EV2 [file 44318_2024_31_MOESM8_ESM.zip › Movie EV2 Legend.docx]

Movie EV2: Timelapse fluorescence microscopy of MDA-MB-157 cells in KIF18Ai

Widefield fluorescence microscopy of MDA-MB-157 H2B-iRFP eGFP-α-Tubulin cell lines in DMSO (left) or KIF18Ai (right)
