# Supplementary figures and images for: Weakened APC/C activity at mitotic exit drives cancer vulnerability to KIF18A inhibition
Source: EMBO J. 2024 Jan 26;43(5):2. doi: 10.1038/s44318-024-00031-6 (PMC10907621; doi:10.1038/s44318-024-00031-6)

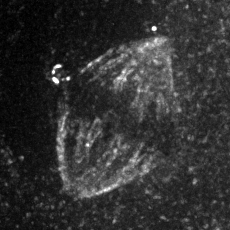

Supplement: Supplementary file 12 — Source Data Fig. 1 [file 44318_2024_31_MOESM12_ESM.zip › Figure 1/1A/OVCAR-3_DMSO_488-aTub_555-KIF18A_647-CENPA.tif]

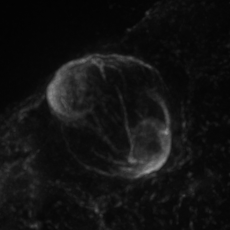

Supplement: Supplementary file 12 — Source Data Fig. 1 [file 44318_2024_31_MOESM12_ESM.zip › Figure 1/1A/OVCAR-3_KIF18Ai_488-aTub_555-KIF18A_647-CENPA.tif]

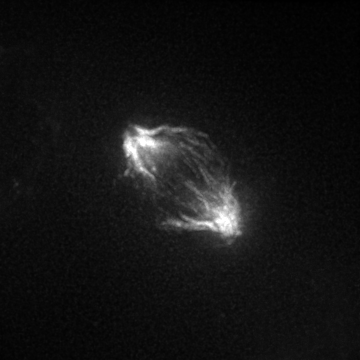

Supplement: Supplementary file 13 — Source Data Fig. 2 [file 44318_2024_31_MOESM13_ESM.zip › Figure 2/2H/HCC1806_WT_KIF18Ai_488-tub_555-BubR1_647-MAD1_DAPI.tif.tif]

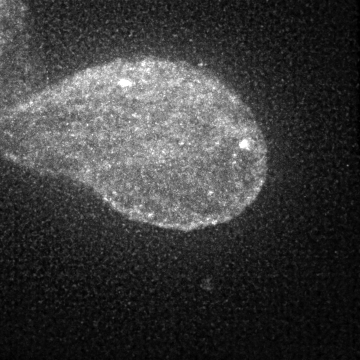

Supplement: Supplementary file 13 — Source Data Fig. 2 [file 44318_2024_31_MOESM13_ESM.zip › Figure 2/2H/HCC1806_WT_Nocodazole_488-tub_555-BubR1_647-MAD1_DAPI.tif]

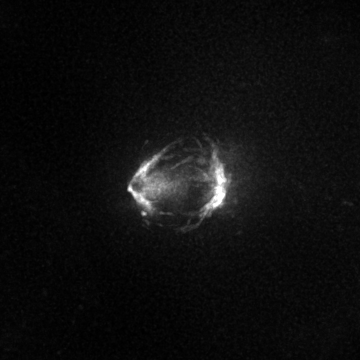

Supplement: Supplementary file 13 — Source Data Fig. 2 [file 44318_2024_31_MOESM13_ESM.zip › Figure 2/2H/HCC1806_WT_DMSO_488-tub_555-BubR1_647-MAD1_DAPI.tif]

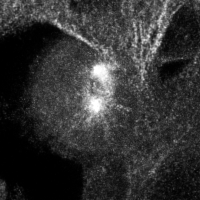

Supplement: Supplementary file 14 — Source Data Fig. 3 [file 44318_2024_31_MOESM14_ESM.zip › Figure 3/3E/HCC1806_KIF18Ai_HighResMitosis.tif]

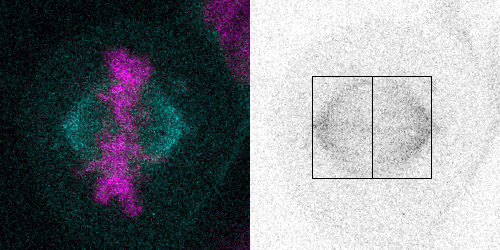

Supplement: Supplementary file 14 — Source Data Fig. 3 [file 44318_2024_31_MOESM14_ESM.zip › Figure 3/3F/HCC1806_DMSO_PA-GFP-Tub.tif]

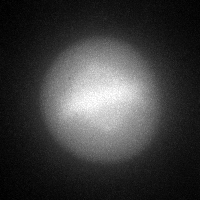

Supplement: Supplementary file 17 — Source Data Fig. 6 [file 44318_2024_31_MOESM17_ESM.zip › Figure 6/6E/HeLa-CyB-eYFP_DMSO.tif]

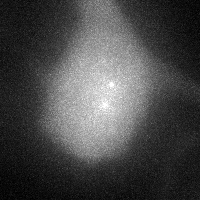

Supplement: Supplementary file 17 — Source Data Fig. 6 [file 44318_2024_31_MOESM17_ESM.zip › Figure 6/6E/RPE1-CyB-eYFP_DMSO.tif]
