# Supplementary material for: Weakened APC/C activity at mitotic exit drives cancer vulnerability to KIF18A inhibition
Source: EMBO J. 2024 Jan 26;43(5):2. doi: 10.1038/s44318-024-00031-6 (PMC10907621; doi:10.1038/s44318-024-00031-6)
Supplement: Supplementary file 19 — Expanded View Figures [file 44318_2024_31_MOESM19_ESM.pdf]

Expanded View Figures

Figure EV1. Extended analysis of cellular response to KIF18Ai.

(**A**) Wide-field immunofluorescence of KIF18A localization relative to spindle poles (CEP192, yellow) and the mitotic spindle (α-Tubulin, magenta) in response to KIF18Ai treatment across full cell line panel. Scale bar = 5 μm. (**B**) Titration of KIF18Ai in a 5-day MTT endpoint viability assay for the panel of sensitive and insensitive cell lines. $N \geq 3$ independent experiments, $n \geq 2$ technical replicates per experiment. Data are represented as mean ± SEM. (**C**) Western blot of KIF18A expression levels across the full panel of sensitive and insensitive cell lines. (**D**) Linear correlation between 5-day KIF18Ai toxicity and normalized KIF18A expression (KIF18A/α-Tubulin) from (**C**). Expression is plotted on a $\log_2$ axis. (**E**) Linear correlation between 5-day KIF18Ai toxicity and Nutlin-3 toxicity from a 5-day MTT endpoint viability assay for the panel of sensitive and insensitive cell lines. $N = 3$ independent experiments, $n = 3$ technical replicates per experiment. Data are represented as mean ± SD.

 © The Author(s)

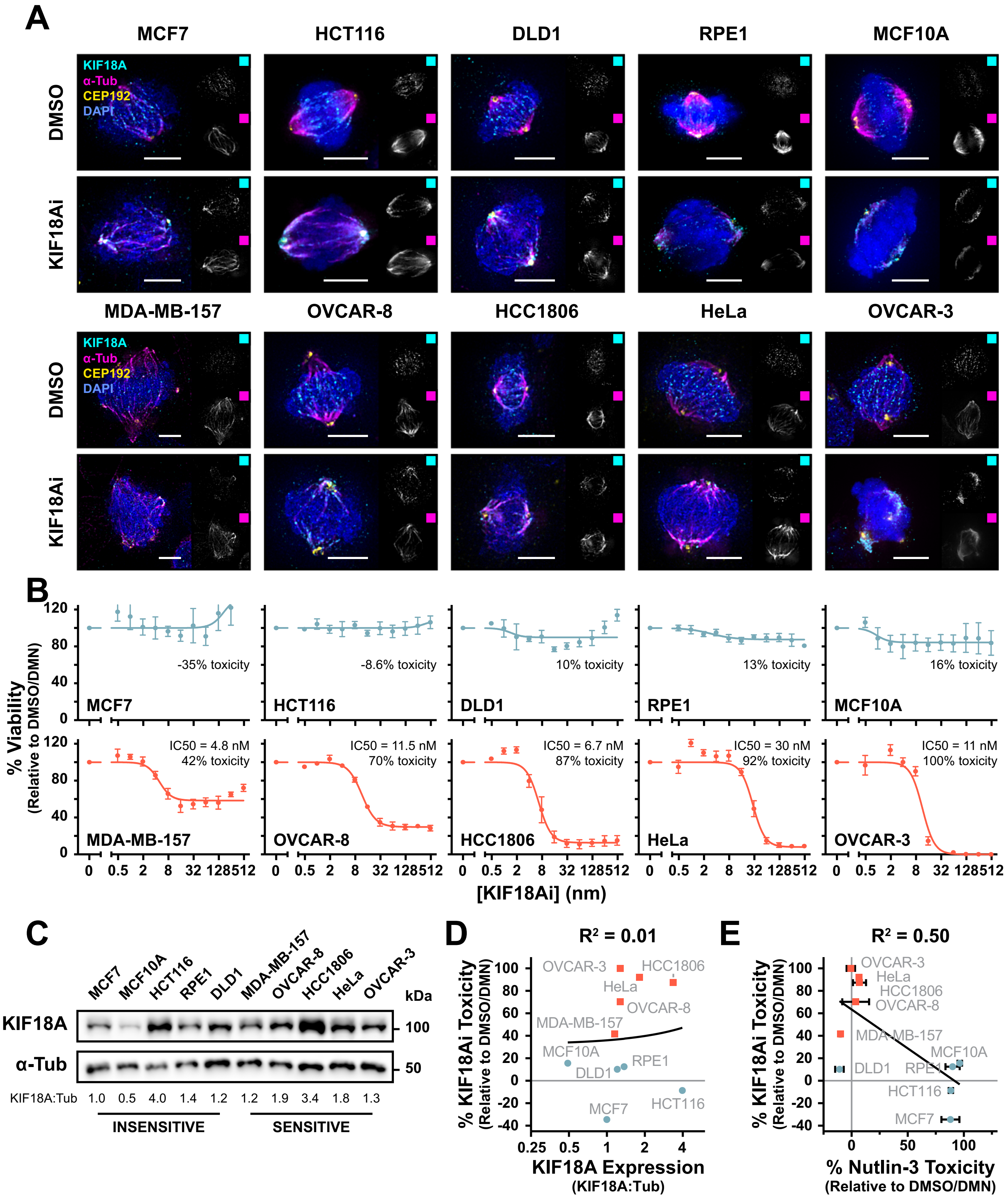
A
MCF7
HCT116
DLD1
RPE1
MCF10A
DMSO
KIF18Ai
KIF18A
α-Tub
CEP192
DAPI
MDA-MB-157
OVCAR-8
HCC1806
HeLa
OVCAR-3
B
% Viability
(Relative to DMSO/DMN)
-35% toxicity
-8.6% toxicity
10% toxicity
13% toxicity
16% toxicity
IC50 = 4.8 nM
42% toxicity
IC50 = 11.5 nM
70% toxicity
IC50 = 6.7 nM
87% toxicity
IC50 = 30 nM
92% toxicity
IC50 = 11 nM
100% toxicity
0 0.5 2 8 32 128 512
[KIF18Ai] (nm)
C
MCF7 MCF10A HCT116 RPE1 DLD1 MDA-MB-157 OVCAR-8 HCC1806 HeLa OVCAR-3
kDa
KIF18A
α-Tub
100
50
KIF18A:Tub 1.0 0.5 4.0 1.4 1.2 1.2 1.9 3.4 1.8 1.3
INSENSITIVE
SENSITIVE
D
R^2 = 0.01
% KIF18Ai Toxicity
(Relative to DMSO/DMN)
KIF18A Expression
(KIF18A:Tub)
E
R^2 = 0.50
% Nutlin-3 Toxicity
(Relative to DMSO/DMN)

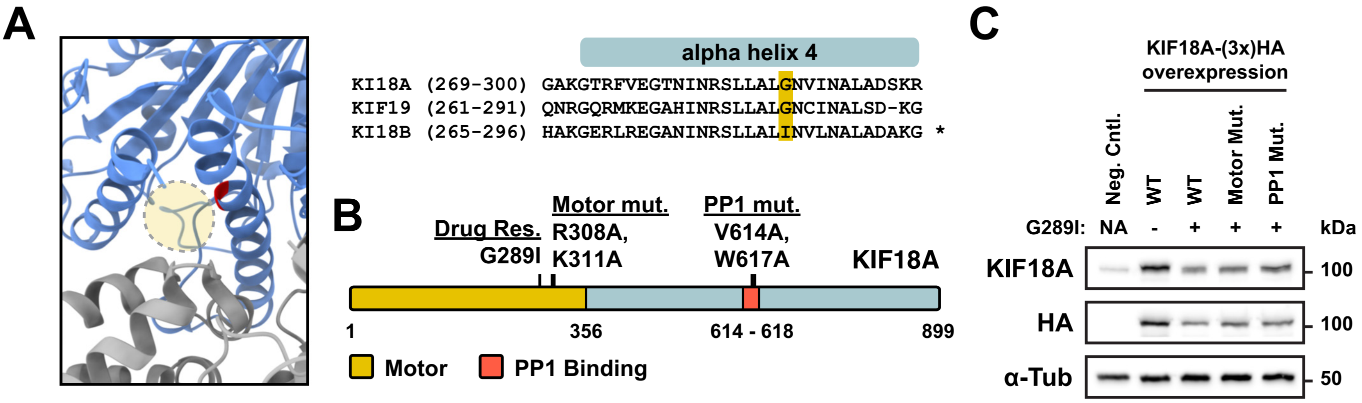

Figure EV2. Validation of drug-resistant KIF18A transgenic HCC1806 cells.

(**A**) Schematic of KIF18Ai drug binding site from EM structure (PDB: 5OAM). Blue: KIF18Ai motor domain with G289 highlighted in red. Gray: α-tubulin/β-tubulin. Highlighted circle represents drug binding pocket. Protein sequence alignment of drug binding pocket between KIF18A, KIF19, and KIF18B. (**B**) Schematic of KIF18A protein domains and mutations. (**C**) Western blot validation of HCC1806 cells constitutively expressing a WT, drug-resistant (G289I), drug-resistant and motor dead (G289I, R308A, K311A), or drug-resistant and PP1-binding deficient (G289I, V614A, W617A) KIF18A-(3x)HA transgene.

 © The Author(s)

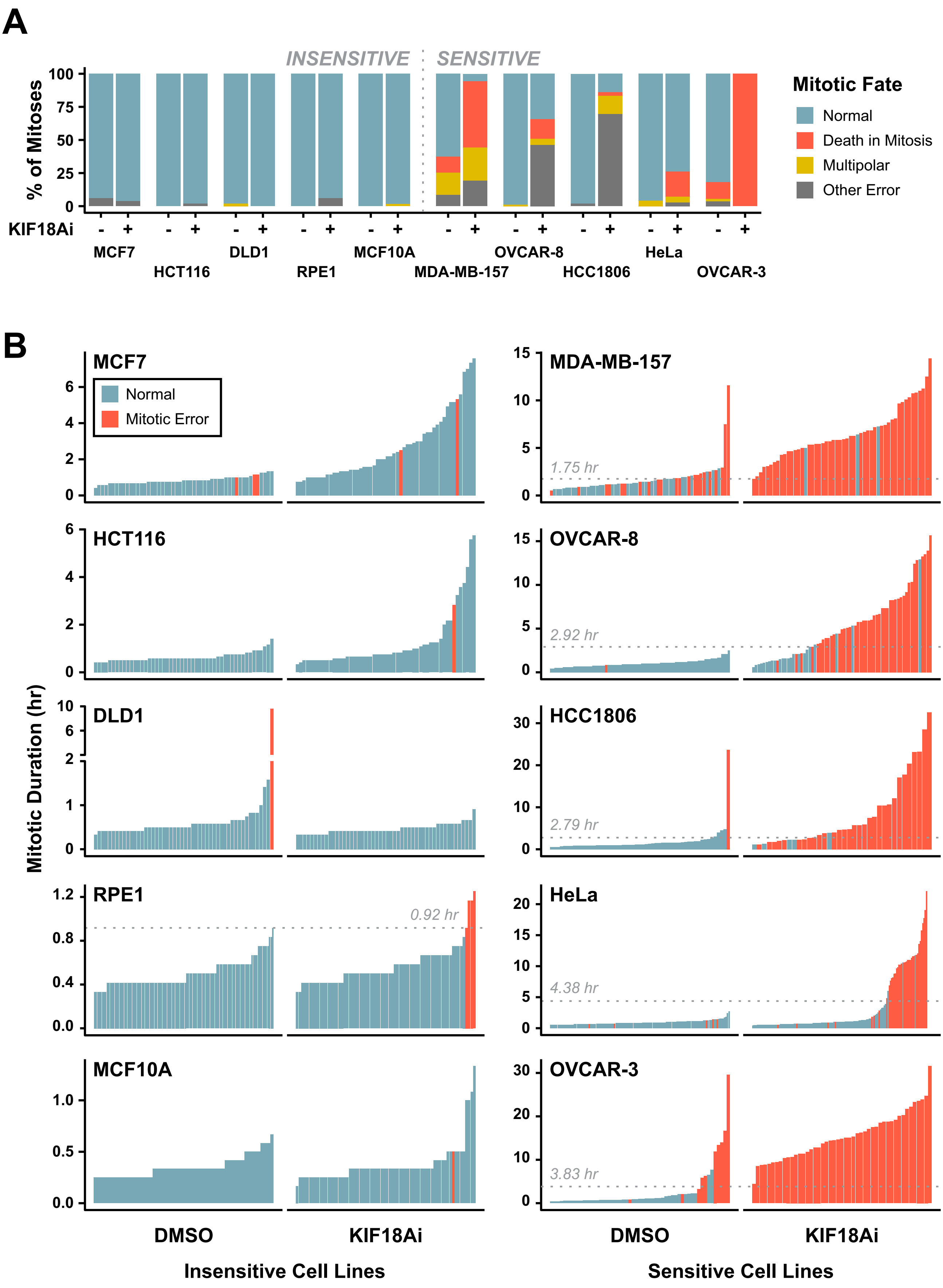
A
INSENSITIVE
SENSITIVE
% of Mitoses
100
75
50
25
0
KIF18Ai
MCF7
HCT116
DLD1
RPE1
MCF10A
MDA-MB-157
OVCAR-8
HCC1806
HeLa
OVCAR-3
Mitotic Fate
Normal
Death in Mitosis
Multipolar
Other Error
B
Mitotic Duration (hr)
Mitotic Error
1.75 hr
2.92 hr
2.79 hr
0.92 hr
4.38 hr
3.83 hr
DMSO
KIF18Ai
Insensitive Cell Lines
Sensitive Cell Lines

Figure EV3. Live-cell analysis of mitotic outcomes from KIF18Ai Treatment.

(**A**) Proportion of mitotic fates across the panel of H2B/α-Tubulin fluorescently tagged cell lines in response to DMSO or KIF18Ai treatment from live-cell wide-field time-lapse microscopy in Fig. 1E. (**B**) Representation of mitotic error threshold across a panel of H2B/α-Tubulin fluorescently tagged cell lines in response to DMSO or KIF18Ai treatment from live-cell wide-field time-lapse microscopy in Fig. 1E. Bars represent individual mitotic events. Dotted line represents error threshold.

© The Author(s)

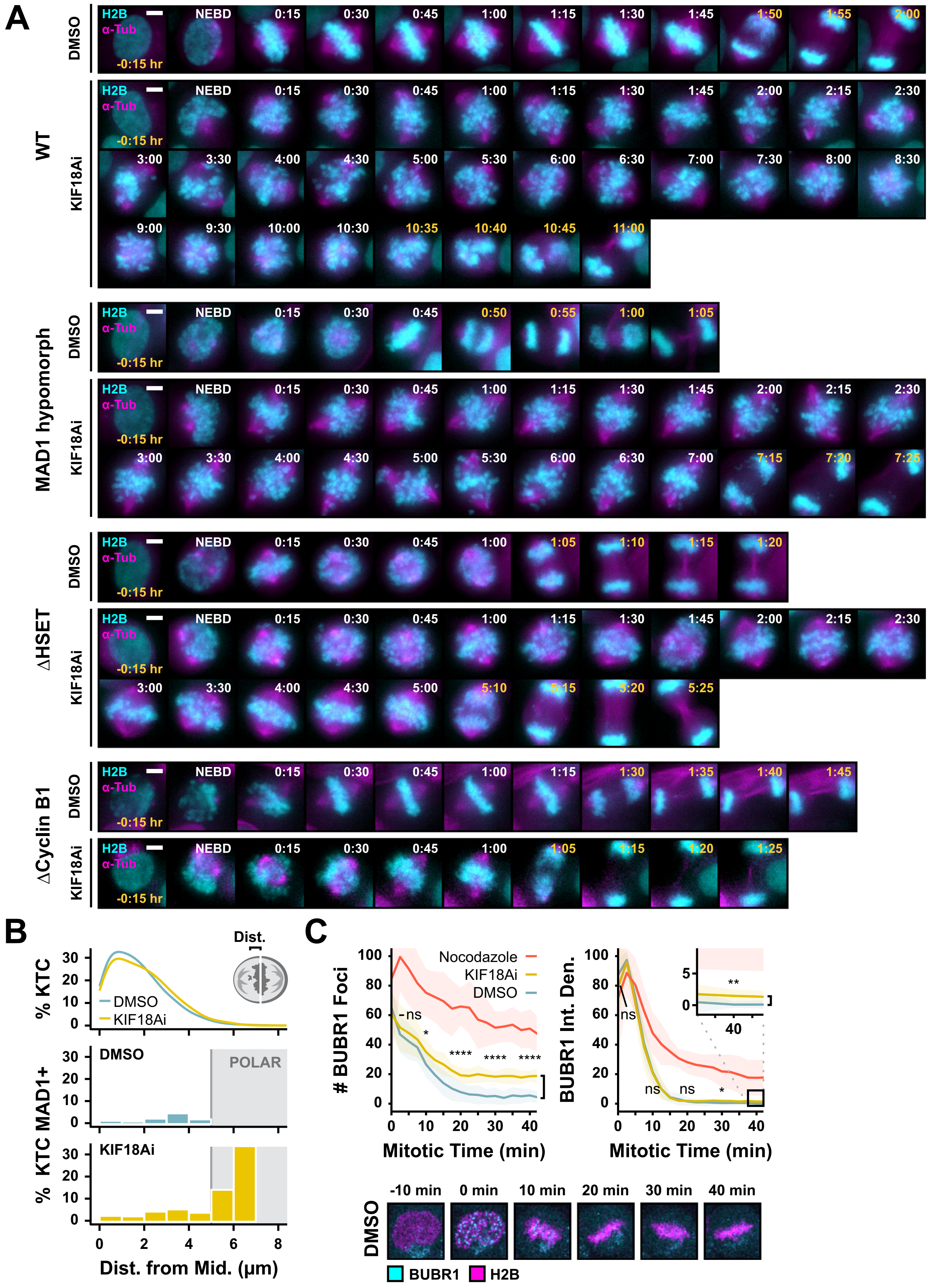
A
WT
DMSO
KIF18Ai
MAD1 hypomorph
ΔHSET
ΔCyclin B1
H2B
α-Tub
-0:15 hr
NEBD
B
% KTC
Dist.
DMSO
KIF18Ai
% KTC MAD1+
POLAR
Dist. from Mid. (μm)
C
Nocodazole
BUBR1 Foci
BUBR1 Int. Den.
Mitotic Time (min)
ns
-10 min
0 min
10 min
20 min
30 min
40 min
BUBR1
H2B

Figure EV4. Extended chromosome alignment and SAC activation data in KIF18Ai.

(**A**) Representative live-cell time-lapse microscopy stills from HCC1806 cells used to evaluate mitotic chromosome congression. Scale bar = 5 μm. (**B**) Top: Kinetochore distribution probability along the spindle axis from Fig. 3C. Bottom: Probability of a kinetochore being MAD1+ relative to its position on the spindle axis in DMSO or KIF18Ai conditions. Polar kinetochores are defined as >5 μm from the spindle midline. (**C**) Top: Quantification of BUBR1 foci from live-cell confocal time-lapse microscopy of HeLa EGFP-BUBR1 BAC H2B-iRFP cell lines in DMSO, KIF18Ai, and Nocodazole conditions. Error bars represent mean ± SD. Statistical significance was determined using an unpaired two-tailed Student's *t* test between DMSO and KIF18Ai conditions at 0, 10, 20, 30, and 40 min. Sample size and full statistical results are listed in Dataset EV2. Bottom: Representative still images from mitotic movies. *P < 0.05, **P < 0.01, ***P < 0.001, and ****P < 0.0001.

 © The Author(s)

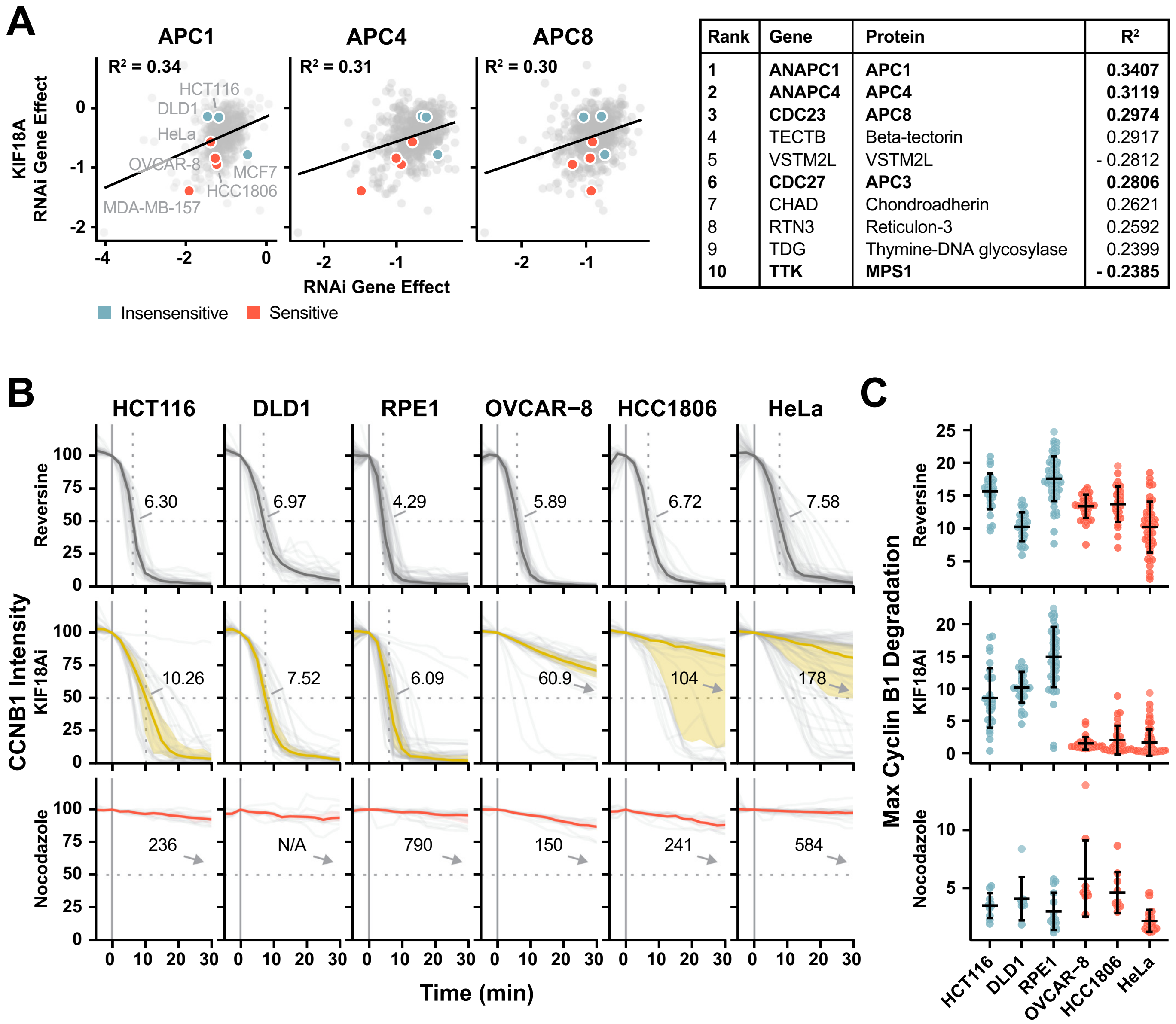

Rank	Gene	Protein	R^2
**1**	**ANAPC1**	**APC1**	**0.3407**
**2**	**ANAPC4**	**APC4**	**0.3119**
**3**	**CDC23**	**APC8**	**0.2974**
4	TECTB	Beta-tectorin	0.2917
5	VSTM2L	VSTM2L	- 0.2812
**6**	**CDC27**	**APC3**	**0.2806**
7	CHAD	Chondroadherin	0.2621
8	RTN3	Reticulon-3	0.2592
9	TDG	Thymine-DNA glycosylase	0.2399
**10**	**TTK**	**MPS1**	**- 0.2385**

Figure EV5. Extended analysis of APC/C activity relative to KIF18A dependency.

(**A**) Top 10 RNAi co-dependency relationships from DepMap dataset for KIF18A. Red and blue points represent sensitive and insensitive cell lines respectively from the panel in Fig. 1C. Bolded table entries are APC/C or SAC genes. (**B**) Quantification of Cyclin B1 degradation rates for endogenously tagged fluorescent cells treated with Reversine, KIF18Ai, or Nocodazole. The median (colored line) of individual traces (gray lines) is plotted, with the shaded region encompassing the first and third quartile of each population. Cyclin B1 signal is normalized to the metaphase inflection point and median $t_{1/2}$ values are listed. Full sample size information is listed in Dataset EV3. (**C**) Maximum slope of Cyclin B1 degradation at metaphase for endogenously tagged fluorescent cells treated with Reversine, KIF18Ai, or Nocodazole in (**B**). Rates are calculated relative to the total Cyclin B1 signal at mitotic entry. Data are represented as mean ± SD. $N \geq 20$ cells per condition (KIF18Ai, Reversine), $N \geq 5$ cells per condition (nocodazole).

© The Author(s)
